# Experiences, barriers and enablers of stroke end-of-life care: a systematic review with mixed-methods synthesis

**DOI:** 10.1186/s12904-026-02211-z

**Published:** 2026-07-27

**Authors:** Clare Gordon, Catherine Harris, Christopher Burton, Bee Wee, Catherine Davidson, Mehrunisha Suleman, Damian Jenkinson, Nikola Sprigg, Alison Rouncefield-Swales, Andrew Clegg, Caroline L. Watkins, Catherine Elizabeth Lightbody

**Affiliations:** 1 School of Nursing and Midwifery, Brook Building, University of Lancashire, Preston, PR1 2HE UK; 2 Applied Health Research Hub, University of Lancashire, Preston, PR1 2HE UK; 3 School of Health Sciences, University of East Anglia, Norwich, NR4 7TJ UK; 4 Nuffield Department of Medicine, University of Oxford, Old Road Campus, Oxford, OX3 7BN UK; 5 HRH Prince Alwaleed Bin Talal Centre of Islamic Studies, University of Cambridge, Cambridge, CB3 9DA UK; 6 University Hospitals Dorset, Royal Bournemouth Hospital, Bournemouth, BH7 7DW UK; 7 Faculty of Medicine and Health Sciences, University of Nottingham, Nottingham, NG51PB UK

**Keywords:** Palliative Care, Stroke, In-patient, Hospital, Terminal Care, Patient, Family, Systematic Review

## Abstract

**Background:**

Despite advances in stroke care, 12% of patients die within 30 days of stroke highlighting the requirement for high-quality end-of-life care (EoLC) within stroke services. EoLC aims to improve the quality-of-life of patients facing death whilst also supporting their families. EoLC after stroke can be difficult due to complex stroke-specific challenges and little clinical guidance. There is a lack of knowledge and understanding of current provision of poststroke EoLC and the barriers and enablers to quality EoLC. Therefore, the aim of this review was to identify the experiences, expectations, information needs and communication preferences of staff, patients and their families and loved ones regarding hospital-based EoLC after stroke.

**Methods:**

A systematic review and synthesis of research published between 2007–2023, updating and extending the 2007 review by Stevens et al. [1]. Eligibility: primary research studies; reporting the views and experiences of staff, patients and/or their families; EoLC provided to adult hospital patients with ischaemic stroke or primary intracerebral haemorrhage; EoLC provided within 30-days post-stroke; qualitative, mixed method and quantitative studies; published in English from 2007 onwards. MEDLINE, CINAHL, PsycINFO, Cochrane and EMBASE databases were searched up to November 2023.

**Results:**

A total of 28 studies were included. Most were conducted in high-income countries, predominately within hospital based services in Europe (*n* = 19) and North America (*n* = 7). Two studies were conducted in middle-income countries and none in low-income countries. Three studies used mixed methods; 13 qualitative, and 12 quantitative. Most data were from healthcare staff (*n* = 708), four studies included patients’ (*n* = 367) views and experiences and nine studies included *n* = 251 next-of-kin. Data synthesis identified three themes describing the barriers and enablers to the delivery of effective EoLC, which can be used to support improvements in practice: 1. *recognising dying within the context of uncertainty; 2. navigating relationships between staff and families; and 3. the lived experience of the stroke unit environment*.

**Conclusions:**

Stroke unit EoLC is shaped by interconnected dimensions of recognising dying, prognostic uncertainty, and the culture and environment of care delivery. Limited progress in research since 2007, particularly in relation to patient-reported experiences and intervention development, underscores the need for renewed research focus to inform best practice and enhance support for patients and families.

**Trial registration:**

PROSPERO [CRD42020212895].

**Supplementary Information:**

The online version contains supplementary material available at https://doi.org/10.1186/s12904-026-02211-z.

## Background

Despite advances in stroke care in recent decades, stroke-associated mortality remains high. Thirty-day mortality rates are 12 to 40% following a stroke, and 25 to 30%, who initially survive, die within 12 months [2, 3]. Palliative care encompasses EoLC, with EoLC specifically focussed on the end stage of life whereas palliative care has a broader scope aimed at improving quality of life in life-threatening illness [4, 5]. In this review, EoLC refers to the physical, psychosocial, spiritual and emotional care of the person dying, and their families, delivered in the last days and weeks within the context of acute hospital care. We have operationalised this as death within 30 days following stroke, reflecting evidence that up to 40% of patients with acute stroke die within this period and aligning with our focus on hospital-based EoLC [4]. We have used the term ‘family’ to refer collectively to family members, friends, carers, loved ones and next-of-kin of stroke patients.

Stroke presents challenges to the provision of quality EoLC. There are two distinct trajectories of dying following a stroke [6]. In the first, death occurs quickly and suddenly, soon after a stroke. The second trajectory is associated with much uncertainty and may extend for weeks or months, with periods of recovery and decline before eventual death. These trajectories are different from those conditions on which generic models of EoLC were developed such as cancer where, in most cases, trajectories are better understood and death is less sudden, giving time for support and planning around future EoLC wishes [7]. Recognising when to initiate EoLC after stroke is challenging when prognosis is uncertain [5]. These difficulties can cause delays in starting EoLC, resulting in lost opportunities for support, to enhance quality of life and avoid treatments which may be futile or invasive [8].

In the context of hospital-based stroke care, staff are expected to deliver EoLC within a culture and environment heavily focussed upon recovery. Generic EoLC guidance and tools do not consider the range of complex condition-specific challenges to the delivery of quality EoLC that stroke presents [9]. These include the sudden onset of stroke; prognostic uncertainty; communication and cognitive difficulties; low levels of consciousness; psychosocial problems; and other stroke-specific symptoms [10]. These create difficulties for planning care, and when patients are unable to communicate their wishes, a greater emphasis on family involvement in decision-making, who may be inadequately prepared [10]. Stevens et al. [1] reviewed evidence to identify the EoLC needs of stroke patients. However, hospital stroke care has evolved substantially since 2007, driven by the expansion of hyperacute interventions (e.g. thrombectomy and neurosurgery) and the introduction of hub‑and‑spoke models of stroke service organisation. These developments have increased both the range of medical interventions available and the frequency of patient transfers between clinical teams. This previous review did not explore the barriers and enablers to the delivery of quality EoLC and what the key issues are to address quality improvement.

### Aims of the review

This mixed methods systematic review aimed to update and extend a previous review conducted in 2007 by Stevens et al. [1] and critically evaluate the current international evidence on the experiences of patients, their families and staff, and the barriers and enablers to quality EoLC for stroke patients in hospital. Specifically, the review objectives are to identify:

- The experiences, expectations, satisfaction levels, information needs and communication preferences of staff, stroke patients and their families regarding hospital-based post-stroke EoLC, and the specific factors that influence these.
- The barriers and enablers to delivering effective hospital-based post-stroke EoLC, from the views of staff, patients and their families.

## Methods

This systematic review, prospectively registered on PROSPERO CRD42020212895 [11], used the Joanna Briggs Institute (JBI) guidance for mixed methods systematic reviews [12] and is reported according to the PRISMA 2020 statement [13].

### Search strategy

We searched the following databases on the 29th November 2023: MEDLINE (Ovid), CINAHL (EBSCOhost), PsycINFO (EBSCOhost), Cochrane Library all databases (via Wiley) and EMBASE (Ovid). A comprehensive search strategy developed in conjunction with an Information Specialist (CH), was used. Search terms were related to cerebrovascular disorders, stroke, palliative care, end-of-life and bereavement. Search terms for stroke were taken from a published Cochrane Stroke Gorup search strategy [14]. Given the interchangeable use of terminology to define death and dying in the acute stroke phase, we searched a broad range of terms to identify relevant studies that may not be explicitly labelled as EoLC. Full details of the search strategy are provided in Additional file 1.

The search was limited to records published between 2007 and the search date, to identify studies published after the review by Stevens et al [1]. We screened reference lists of all included studies and the seven studies from Stevens et al.’s [1] review.

### Inclusion and exclusion criteria

We accepted the author’s definition of EoLC (if it met the 30 days post-stroke eligibility) and acute stroke. Studies were eligible for inclusion if the: (1) full text was available in English; (2) the paper presented empirical data; (3) EoLC was provided to patients with ischaemic stroke or primary intracerebral haemorrhage; (4) EoLC was delivered in hospital and within 30-days post-stroke (5) the data reported views of staff, patients and/or their families.

We excluded papers reporting only process data, audits, case studies, conference abstracts, opinion or discussion papers, reviews, letters, editorials and grey literature. To reduce heterogeneity and focus the review on experiences within stroke service delivery, we also excluded patients with subarachnoid haemorrhage as they typically receive hospital care outside of specialist stroke services.

### Selection and data extraction

Search results were imported into Endnote for deduplication. Titles, abstracts and full texts were screened independently by two reviewers (LW, CD, CT, CG & CEL). Discrepancies were resolved through team discussion. Authors were contacted for stroke data when studies reported mixed-conditions, or to clarify published data. Data extraction was completed by researchers NL, ARS, CT, CG and CEL and verified by a second reviewer.

As this review includes qualitative and quantitative data, separate extraction methods were developed and piloted. For mixed methods studies, we used separate extraction forms for qualitative and quantitative components. We extracted participant and intervention characteristics, as well as barriers, enablers, experiences, satisfaction, expectations, information needs and communication preferences of staff, stroke patients, and families.

#### Quantitative data extraction

To accommodate the heterogeneity in methods and outcomes measures, we adopted a broad approach to data extraction. Where available, we extracted: (a) descriptive statistics; (b) correlations; (c) key findings within the results sections that were relevant to the review objectives.

#### Qualitative data extraction

We extracted data in relation to the review objectives, including: (a) contextual information; (b) empirical findings and (c) authors’ interpretations. Empirical data were extracted verbatim to preserve the original meaning and context.

### Assessment of risk of bias in included studies

Included studies were independently assessed using the Mixed Methods Appraisal Tool (MMAT) [15] and verified by a second reviewer. Studies were rated using a percentage scoring system which resulted in the assignment of a star rating from 5* to 1*, with 5* being the highest and 1* the lowest. No studies were excluded based on quality, though the team considered the study limitations when conducting data synthesis and using data in studies which received a lower quality rating.

### Data synthesis

We followed a convergent integrated approach to data synthesis and integration, in which findings from qualitative and quantitative studies were combined to generate a unified set of interpretive themes representing shared meanings across data sources [12]. Quantitative data were transformed into qualitative form (qualitizing) through narrative interpretation. These qualitized data, alongside data extracted from qualitative studies, were uploaded to NVivo 12 [16] for integrated analysis. Reviewers independently categorised the data by similarity of meaning and through constant comparison, grouping categories of shared meaning [12, 17]. Reviewers (AR, NL, CD, CT, CG) subsequently compared categories, with disagreements resolved through discussion; categories were iteratively developed into themes and discussed with the wider team (CEL, CT, CG & CW) until agreement was reached. We further examined the extent to which the inclusion of lower-quality studies influenced the derived categories and themes.

## Results

**Fig. 1:**
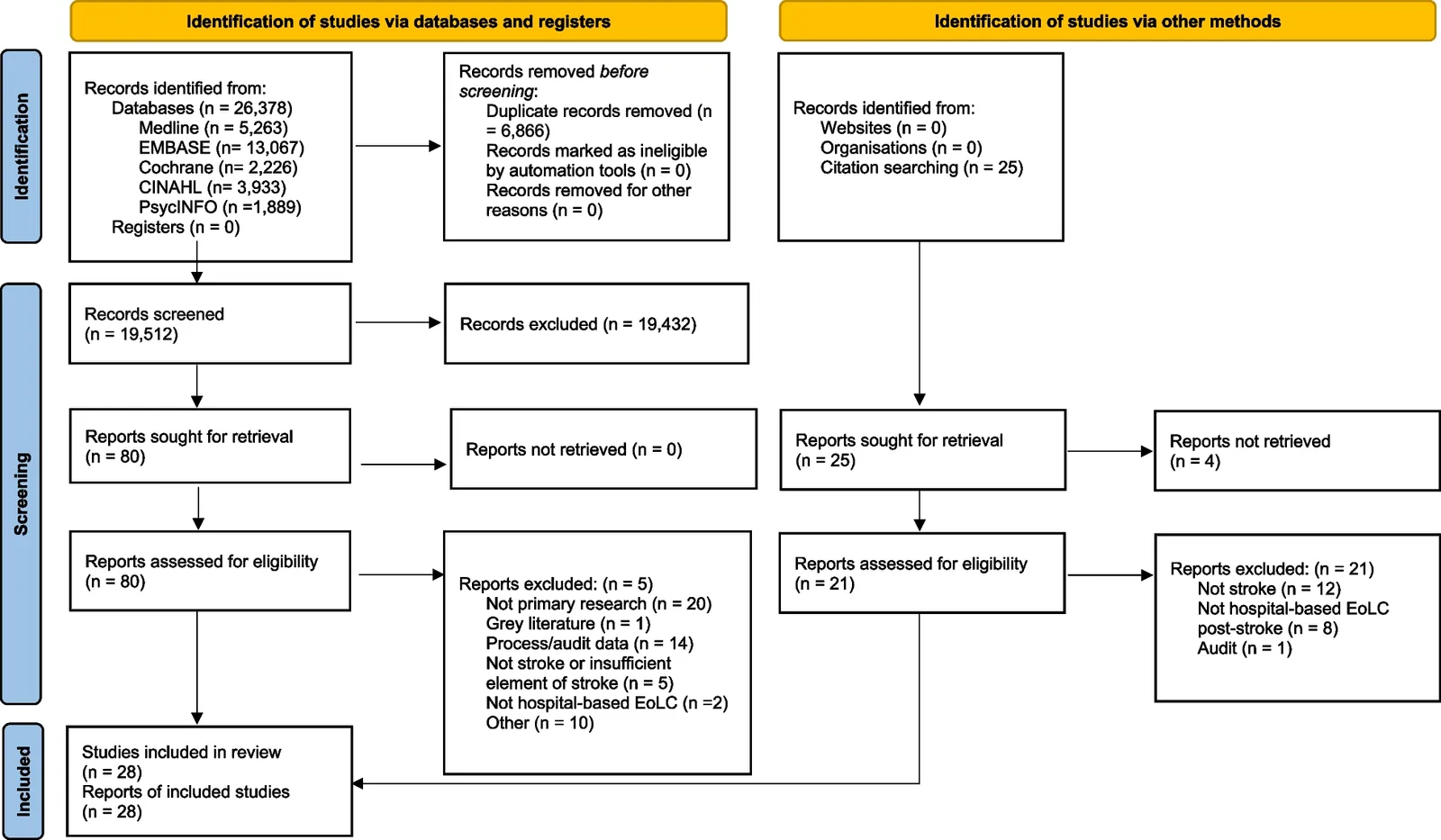
PRISMA flow diagram

The search identified 26,378 papers. After removing duplicates and screening titles and abstracts, 101 full-text papers were assessed for eligibility. Of these, 28 papers met the inclusion criteria, including one paper from Stevens et al.’s [1] review. The PRISMA flow diagram is presented in Fig. 1.

### Overview of included studies

Of the 28 included studies: 3 used mixed methods [18–20]; 13 qualitative methods [8, 10, 21–31] and 12 quantitative methods [32–43]. Most studies originated from high income country settings, with the exception of two [23, 30].

Participants included staff [8, 18, 22–31, 36, 37, 41, 43], families [21, 25, 33, 38, 39], stroke patients [32, 34, 42], combinations of families and staff [19, 29, 35, 40] or families and stroke patients [10]. In total, *N* = 708 staff, *N* = 251 families and *N* = 367 patients were included in this review. An additional file provides detail on the study characteristics (see Additional file 2—Table A1 Summary table of included studies).

Qualitative studies employed a range of data collection methods, including individual or focus group interviews [10, 21, 23, 25, 26, 28–31], focus groups [24], non-participant observation [31], secondary analysis of interview studies [29] and combined interviews with focus groups and a qualitative survey [8, 22].

The three mixed methods studies [18–20] used interviews and focus groups for the qualitative components. Quantitative data was collected through surveys and medical case note review.

Quantitative methods included: retrospective chart reviews [32]; prospective clinical studies [34]; pilot randomised controlled trials [35]; fictional vignette cases [41]; and quantified responses from questionnaires and structured interviews.

### Quality appraisal

**Fig. 2:**
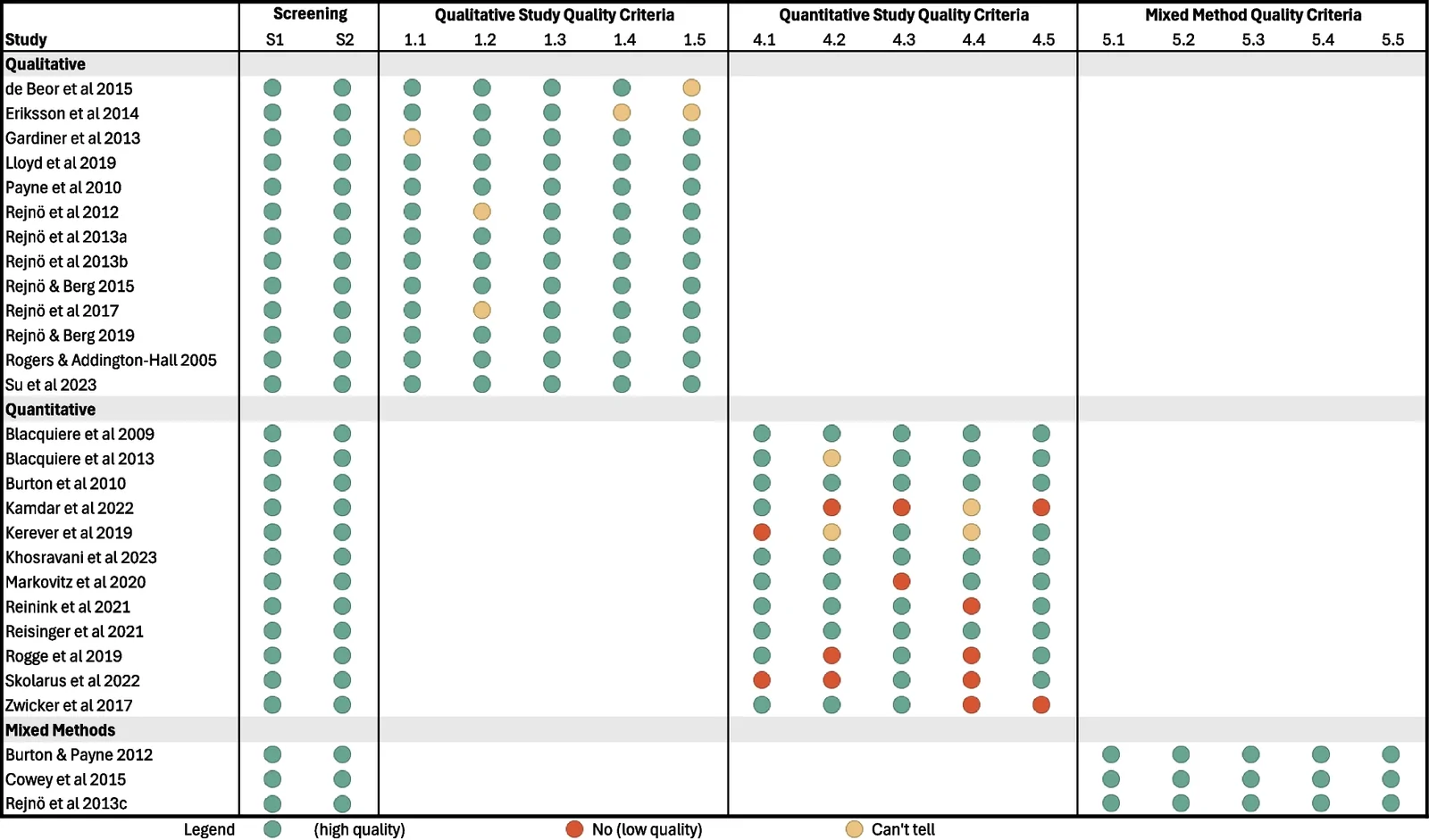
Visual summary of quality appraisal using MMAT

The Mixed Methods Appraisal Tool (MMAT) [15] was used to assess the methodological quality of the studies and was chosen as it can appraise different study designs. The results of the quality assessment are presented in Fig. 2. Overall, the quality of evidence was reasonably good (Table 1). Quantitative studies were of more variable quality.

**Table 1:** Overview of quality of evidence according to study type

	5* (100%)	4* (80%)	3* (60%)	2* (40%)	**Total**
Qualitative	8	4	1	0	13
Quantitative	4	3	3	2	12
Mixed methods	3	0	0	0	3
Total	14	7	4	2	28

### Synthesis of included studies

#### Experiences and influences of hospital EoLC

Although the experiences of patients, families and staff differed, three overarching themes shaped their experiences of post-stroke EoLC:

1. Recognising dying in the context of prognostic uncertainty
2. Navigating relationships between staff and families
3. The lived experience of the stroke unit environment.

**Fig. 3:**
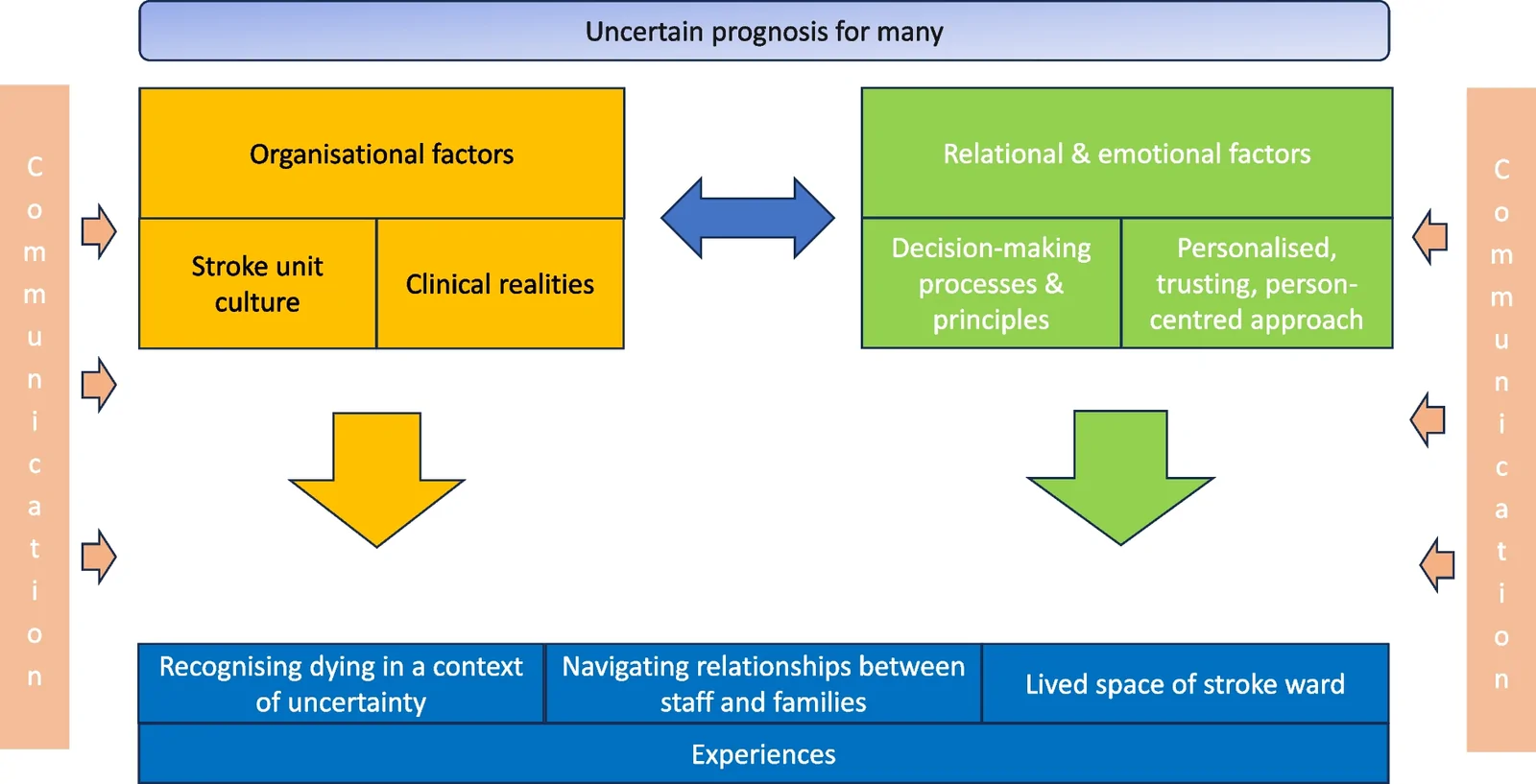
Diagrammatic representation of inter-relationships between the main themes

While presented as distinct, these themes are interrelated and layered with complexity. Figure 3 illustrates the themes and their interconnections. Each theme is influenced by a range of factors, which may act as barriers or enablers to the provision of high-quality EoLC following stroke.

#### Theme 1—Recognising dying in the context of prognostic uncertainty

Drawing on staff and family perspectives, uncertainty of prognosis after stroke underpins many of the challenges to timely and high-quality EoLC identified in this review. The timing of limiting life-sustaining treatment is important for both physicians and nurses [40]. However, accurately recognising the time point at which patients require palliation is challenging, particularly early after stroke [8, 18, 19, 37, 40]. Some teams may seek to postpone treatment decisions in the hope that the patient’s condition will become clearer, or more information is available, over time [22, 24, 40]; but this can lead to overtreatment [8, 22] and missed opportunities for timely EoLC [8, 10, 18, 19, 34]. These challenges are compounded when patients are unable to communicate their wishes, requiring healthcare teams to seek a second opinion, or rely on families to convey patient wishes [24, 25, 29, 36, 43].

Balancing active treatment and supportive care is often difficult, especially as the patient’s condition changes over time. Families may struggle to understand the shifting prognosis and reasons behind changes in care [22, 24, 26]. This uncertainty can make decision-making particularly difficult for families [10, 21, 22, 24, 32, 36]. In this context, staff play a crucial role in managing families’ expectations [19, 20, 25, 26, 32] and reinforcing the inherent uncertainty of prognosis [10, 18, 20, 21, 23, 25, 30, 35, 37].

Together, the difficulties for staff to recognise when a patient is at the end-of-life, and for families to contribute to care decisions when there is prognostic uncertainty, leads to complexity in decision-making. This can lead to disagreements and feelings of chaos and insecurity among families, staff and patients themselves, which connects to our second theme on relationships [10, 18, 19, 19, 21, 22, 22, 24, 26, 29, 37].

#### Theme 2—Navigating relationships between staff and families

Again, drawing on staff and family perspectives, this theme draws on the interpersonal nature of relationships between staff and families. Communication was a pivotal factor across all aspects of EoLC experiences, as shown in Fig. 3**.** Effective communication underpins positive staff-family relationships, drawing on an extensive range of staff skills, experience and self-awareness to build relationships [18, 20, 26, 38, 40]. The most reported conflicts between stroke unit staff and families were around cessation of feeding, changing to EoLC and providing comfort [32, 33, 39]. Involvement of palliative care teams supported relationships between physicians and nurses in the same stroke team, and between families and staff providing EoLC [8, 35, 37, 40]. A complex relationship between physicians and nurses is emerging from the data [22, 35]. When physicians involved nurses in EoLC decisions, active treatment was more often withheld [35]. Nurses’ opinions tended to be in-line with the patient’s family and hence heightened the importance physicians placed on family opinions [35, 39]. However, even when involved, nurses generally were less satisfied with EoLC decisions than physicians [27, 40]. There was insufficient data on patients’ experiences of relationships with staff or family members to report on this.

#### Theme 3 – The lived experience of the stroke unit environment

The final theme concerns the lived experience of the stroke unit space, encompassing the realities of everyday actualities that shape the delivery and perception of EoLC for patients, families and staff [20, 33]. For example, in Burton et al.’s [18] study, patient-reported fatigue was seen by staff as exacerbated by environmental factors limiting rest. Organisational structures, routines and cultures within stroke units [8, 10, 20, 22]—including time pressures, workload demands, and staff roles [20, 22, 26, 27, 29] – can all influence the quality and experience of EoLC. These factors not only affect the logistics of care but also infer meaning to different types of care within the stroke unit space, and therefore also the experiences of patients, families and staff [29]. A high degree of importance staff place on EoLC enables them to deliver quality EoLC on busy stroke units [8, 31]. Stroke teams consider specialist palliative care as an essential component within stroke care [8, 22, 31]. However, in one Canadian study, surveyed stroke physicians preferred general palliative care units as they felt palliative care specialists were best positioned to coordinate patients’ EoLC needs [37]. Family satisfaction with care provided on stroke units was generally high [40]. Compared to the first two themes, data on this theme was not reported as explicitly nor as much.

#### Barriers and enablers to delivering EoLC

Each of the themes shaping the experiences of patients, families and staff were influenced by a range of interconnected factors. These can be categorised into: 1. Organisational 2. Relational; and 3. Emotional factors. Many of these factors intersect across the experience themes (see Fig. 3).

### Organisational factors

Organisational barriers included stroke unit cultures focused on recovery, limited staff training and confidence, rigid organisational routines, time pressures and environments lacking space for privacy [8, 10, 18–20, 26, 27, 37, 43]. These practical constraints may lead to staff prioritising acute aspects of care and compromise EoLC to patients and their families [27]. One study reported that no patients were referred for specialist palliative care or offered choices such as early discharge home [10]. The physical environment for EoLC was debated; some families felt the side rooms and private space in hospitals adequate, whereas others felt that this could be isolating [10, 18]. Organisational enablers included early palliative care involvement, co-ordination and collaboration within the stroke team, and discharge planning to enable patients to die out of hospital (e.g. at home) [8, 10, 18, 29, 36]. One study found that additional palliative care consultations did not lead to a ‘self-fulfilling prophesy’—they did not lead to increased mortality rates—allaying concerns about premature withdrawal of care [35].

### Relational factors

Staff have a pivotal role in facilitating shared decision-making with patients, families and colleagues. Relational barriers were founded on the need for proxy decision-making due to the patient being unable to communicate their wishes, poor uptake of Advance Directives and family not knowing what the patient would have wanted. This could lead to team disunity, avoidance and breakdown of consistent communication between staff and family. Avoidance may hinder relationship-building, delay decision-making and negatively impact care [10, 18, 20, 23, 24, 38, 43]. Some family members preferred clinicians to make decisions on their behalf [21].

Relational enablers included personalised care, consistency in communication and collaborative decision-making involving the stroke team, family and palliative care specialists. Families play a vital role in helping staff understand the patient as a person and in communicating the patient’s wishes. Honest, ongoing communication fosters security and confidence [27–29]. However, in some cases staff may withhold or pace information-giving to better support families—an approach that depends on strong, trusting relationships [28, 29].

Collaboration in EoLC decision-making emerged as a key enabler across multiple studies [18, 19, 23, 24, 26–29, 36]. When patients or families held strong views about treatment or EoLC, staff may adapt their practice [19, 24, 27, 29] or delay decision-making [26] to foster a sense of unanimity [26, 29]. Collaboration with palliative care specialists provided staff with reassurance and guidance on the decision-making process [18]. Collaborative practices also shaped unit culture – providing a space for discussion and differing perspectives [29], supporting multidisciplinary communication [36], and promoting well-informed decisions [36]. Although formal approaches to EoLC decision-making were associated with benefits such as timely decisions, fewer conflicts [32], and improved identification of patients with EoLC needs [34], these approaches were not often used.

#### Emotional factors

As previously discussed in Theme 1, family were often unprepared after the shock of stroke and needing to contribute to EoLC decisions within the context of prognostic uncertainty [10, 18–21, 24, 26–29]. With limited time to reflect, emotional attachment and hope of survival can dominate family’s contributions to EoLC decisions [10, 20].

Enablers supporting emotional wellbeing included staff providing reassurance and providing time for families to adjust [24]. Whilst staff may modify care to accommodate family preferences or give families more time, staff emphasised that care should remain guided by the patient’s condition and should not unnecessarily prolong dying [26]. Generally, families were also of the same view that treatment should not unnecessarily prolong the dying process [19, 20, 24, 26, 32]. All factors identified are summarised in Table 2.

**Table 2:** Barriers and enablers to EoLC following stroke

Factors	Barriers	Enablers
1. Organisational		
	Stroke unit culture: hyperacute and rehabilitation focus delays EoLC. EoLC is equated with treatment or rehabilitation failure	Integrating of EoLC: EoLC seen as a core component of stroke unit care
	Workforce resources: staff shortages, high workload and lack of time impede responsiveness to EoLC needs	Collaborative culture: MDT meetings to discuss EoLC and interdisciplinary input improves decision-making
	Training: staff lack specialist knowledge and confidence in delivering EoLC with its ethical uncertainty	Responsive care: being responsive to the patient’s condition and prioritising family support and communication
	Environment: Lack of privacy and dignity enabling spaces	Organisational processes: Quiet spaces and supported discharge to home or other settings for EoLC
	Routines: staff organisation, shift patterns hinder responsiveness	Early involvement of palliative care: improves communication, symptom control and emotional support
	Delay in starting EoLC and referral to palliative care	
	Societal norms: in some cultures, discussing dying is taboo, affecting EoLC decisions	
2. Relational		
	Lack of patient voice: poor uptake of Advance Directives or lack of understanding of patient wishes by the surrogate decision-maker	Personalised care: using family insights to inform care decisions
	Communication breakdowns: inconsistent, unclear or unheard information from staff reduces family trust	Consistent, honest communication, that considers how confident family members are around surrogate decision making, supports trust and EoLC decisions
	Team disunity: lack of agreement or consensus among nurses and physicians complicates EoLC	Team collaboration: Dedicated time to discuss EoLC as a team, with process to seek advice/opinions from others
	Family involvement: preference to defer to clinicians’ decision-making	Family involvement in MDT discussions
	Perceptual differences in motivations underpinning EoLC decisions	
3. Emotiona**l**		
	Families’ unpreparedness: suddenness of stroke leaves families unprepared to engage with uncertainty of prognosis and EoLC decisions	Emotional support: allowing families time to adjust and frame EoLC as a transition to provide comfort and dignity
	Distress: frustration, anxiety and concern are experiences by healthcare staff and families, most common when there is prolonged dying	Reassurance from staff: reduces family anxiety
	Uncertainty of prognosis: leads to difficulty for families to process EoLC decisions	

## Discussion

This review synthesised available qualitative and quantitative data on the experiences, communication preferences and information needs for EoLC after stroke in hospital-based settings only. Consistent with Stevens et al.’s [1], the evidence base continues to be dominated by studies from high-income countries in Europe and North America. These results provide a summary of the main factors serving as barriers and enablers to the delivery of effective EoLC which can be used to support improvements in practice. The uncertainty of dying in stroke is pervasive and influences the experiences of EoLC for all involved (patients, families and staff). The quality and type of relationships between staff, family and the patient are the foundations for positive EoLC experiences.

There is a notable lack of data around the role of professions other than physicians and nurses in post-stroke EoLC, despite other aspects of stroke care having a multi-professional approach. Challenges around effective and timely decisions may reflect a lack of confidence, education and training of the stroke team to engage in EoLC, and unclear roles for other professions in the stroke team to participate in EoLC decisions and delivery.

Staff require interpersonal skills of openness, counselling and collaboration – interestingly similar skills required for rehabilitation conversations around ongoing and unpredictable recovery and rehabilitation goals [44, 45]. Hence stroke teams are likely to have the interpersonal skills required, which prompts the question as to why staff do not feel confident or able to use these for EoLC. This paper has outlined the factors which influence the experience of EoLC in acute stroke settings, and the ability of staff to provide a flexible and individualised approach. These align with challenges described in wider discussions around person-centred care within the constraints of inpatient settings and resources that tend to prioritise patients’ biomedical needs [46]. These constraints are further compounded by the common need to make proxy life-limiting care decisions in the context of uncertain prognosis and different motivations behind care decisions.

The current direction of stroke care prioritises saving lives, hyper-acute interventions and improving recovery [47, 48]. This prioritisation of active treatment and recovery is mirrored in the relative paucity of EoLC guidance, particularly when contrasted with the extensive guidance addressing other domains of stroke care [49–51]. Additionally, there is little evidence and guidance to support and empower staff in providing what is required. In England and Wales, previous NHS stroke strategy quality markers on EoLC have disappeared from stroke-specific policy and have instead been incorporated into generic strategies, including the personalised care agenda [47]. Also, national stroke audit datasets (Sentinel Stroke National Audit Programme- SSNAP [3]) have minimal data captured around EoLC practice. This reinforces low prioritisation of EoLC within stroke service developments. Recognising dying, and transitioning from recovery and rehabilitation to EoLC in a balanced and measured way, requires insight and experience into the potential for long-term recovery and quality of life for those who have had serious stroke. This requires clinicians with experience of life after stroke to inform the decision-making process, and insight into the influences of the context of care on decision-making. As in our updated review, Stevens et al.’s [1] review findings also highlighted gaps in stroke professionals knowledge and skills for managing prognostic uncertainty, compounded by the absence of stroke-specific EoLC guidance, with implications for the consistency and quality of EoLC.

### Limitations

Several limitations should be acknowledged when drawing conclusions from our results. The absence of studies from low-income countries and exclusion of no-English papers limits the transferability of findings, particularly in those with different cultural norms around death. Quantitative studies were of variable methodological quality with significant heterogeneity between studies resulting in an inability to conduct meta-analysis. Qualitative studies also varied in quality; most were single centre studies with data generation reliant on participants’ recall. The evidence is strongly weighted towards staff perspectives and, whilst acknowledging the challenges with collecting patient experiences around EoLC, only four studies collected data directly from patients. This weighting of evidence leads to EoLC predominately informed by staff views, though the views of family members provide some insight. Finally, although the review reported on all types of EoLC experience after stroke, overall, the available evidence suggests that experiences are negative. It may be that the high quantity of data on negative experiences was influenced by the researcher’s focus, framework or questioning style. It is though, essential that learning is developed based on both negative and positive experiences to improve future care. Most of the included studies focussed upon patients who died, rather than those who had serious stroke and an uncertain prognosis. Consequently, the evidence does not sufficiently explore the experiences of those patients who would benefit from early EoLC discussions and provisions, but who go on to survive.

## Conclusions

This review found that EoLC experiences were framed within the context of uncertainty of prognosis after stroke and the acute stroke unit culture and environment in which decisions and EoLC were made. This impacted staff and families’ preparedness to engage with EoLC discussions. There were tensions in relationships between staff, patients and families that required skilful navigation through open and honest communication, collaboration, flexibility, and a personalised approach. However, conclusions regarding patients’ experiences should be interpreted cautiously, as they are based on limited patient‑reported data and are largely inferred from family and healthcare staff accounts. Findings also indicate that clinicians could be supported through stroke‑specific education focused on managing prognostic uncertainty and recognising dying, alongside clearer guidance to support transitions between active treatment and end‑of‑life care. Greater involvement and role clarity across the multidisciplinary stroke team, combined with organisational support for difficult conversations and proxy decision‑making, may further enhance clinician confidence. Future research will explore these areas to inform the development and evaluation of targeted, stroke‑specific interventions and models of care to support high‑quality EoLC following stroke.

## Supplementary Information

Additional file 1. Search Strategies. Search strategies and search terms for each database.

Additional file 2. Table A1 Summary table of included studies. A table summarising study characteristics and key findings, grouped into qualitative, quantitative and mixed methods study type

## Data Availability

The data generated and analysed for this review are included in this paper, its additional files and from the corresponding author.

## Abbreviations

EoLC: End-of-life care
JBI: Joanna Briggs Institute
NHS: National Health Service
SSNAP: Sentinel Stroke National Audit Programme
UK: United Kingdom
USA: United States of America
MMAT: Mixed methods Appraisal Tool
