# Supplementary material for: Experiences, barriers and enablers of stroke end-of-life care: a systematic review with mixed-methods synthesis
Source: BMC Palliat Care. 2026 Jul 27;25:317. doi: 10.1186/s12904-026-02211-z (PMC13689126; doi:10.1186/s12904-026-02211-z)
Supplement: Supplementary file 1 [file 12904_2026_2211_MOESM1_ESM.docx]

**Additional file 1: Search strategies**

**Database: Ovid MEDLINE(R) and Epub Ahead of Print, In-Process, In-Data-Review & Other Non-Indexed Citations and Daily <1946 to November 28, 2023>**

**Date searched: 29^th^ November 2023**

1	Cerebrovascular Disorders/

2	exp Basal Ganglia Cerebrovascular Disease/

3	exp Brain Ischemia/

4	exp Carotid Artery Diseases/

5	exp Intracranial Arterial Diseases/

6	exp "Intracranial Embolism and Thrombosis"/

7	exp Intracranial Hemorrhages/

8	Stroke/

9	exp Brain Infarction/

10	exp Vertebral Artery Dissection/

11	(stroke or cerebrovasc$ or brain vasc$ or cerebral vasc$ or cva$ or apoplex$).tw.

12	((brain$ or cerebr$ or cerebell$ or vertebrobasilar or hemispher$ or intracran$ or intracerebral or infratentorial or supratentorial or MCA or anterior circulation or posterior circulation or basal ganglia) adj5 (isch?emi$ or infarct$ or thrombo$ or emboli$)).tw.

13	((brain$ or cerebr$ or cerebell$ or intracerebral or intracran$ or parenchymal or intraventricular or infratentorial or supratentorial or basal gangli$) adj5 (haemorrhage$ or hemorrhage$ or haematoma$ or hematoma$ or bleed$)).tw.

14	or/1-13

15	Palliative Care/

16	Palliative Medicine/

17	Hospices/

18	Terminal Care/

19	exp Death/

20	dying.tw.

21	end of life.tw.

22	end of days.tw.

23	last days of life.tw.

24	final days of life.tw.

25	last month of life.tw.

26	palliat*.tw.

27	(terminal adj6 care).tw.

28	bereave*.tw.

29	((death or die or died) adj6 (sudden* or unexpected* or unanticipated or 30 days or thirty days or month)).tw.

30	advance care plan*.tw.

31	advance* statement*.tw.

32	(document* adj3 (preference* or wish*)).tw.

33	or/15-32

34	14 and 33

35	limit 34 to (english language and yr="2007 -Current")

**Database: Embase (Ovid) <1974 to 2023 November 28>**

**Date searched: 29^th^ November 2023**

1	cerebrovascular disease/

2	exp basal ganglion hemorrhage/

3	exp brain hematoma/

4	exp brain hemorrhage/

5	exp brain infarction/

6	exp brain ischemia/

7	exp carotid artery disease/

8	cerebral artery disease/

9	exp cerebrovascular accident/

10	exp cerebrovascular malformation/

11	exp intracranial aneurysm/

12	exp occlusive cerebrovascular disease/

13	stroke/

14	stroke unit/

15	stroke patient/

16	(stroke or cerebrovasc$ or brain vasc$ or cerebral vasc$ or cva$ or apoplex$).tw.

17	((brain$ or cerebr$ or cerebell$ or vertebrobasilar or hemispher$ or intracran$ or intracerebral or infratentorial or supratentorial or MCA or anterior circulation or posterior circulation or basal ganglia) adj5 (isch?emi$ or infarct$ or thrombo$ or emboli$)).tw.

18	((brain$ or cerebr$ or cerebell$ or intracerebral or intracran$ or parenchymal or intraventricular or infratentorial or supratentorial or basal gangli$) adj5 (haemorrhage$ or hemorrhage$ or haematoma$ or hematoma$ or bleed$)).tw.

19	or/1-18

20	palliative therapy/

21	hospice/

22	terminal care/

23	dying.tw.

24	end of life.tw.

25	end of days.tw.

26	last days of life.tw.

27	final days of life.tw.

28	last month of life.tw.

29	palliat*.tw.

30	(terminal adj6 care).tw.

31	bereave*.tw.

32	((death or die or died) adj6 (sudden* or unexpected* or unanticipated or 30 days or thirty days or month)).tw.

33	advance care plan*.tw.

34	advance* statement*.tw.

35	(document* adj3 (preference* or wish*)).tw.

36	or/20-35

37	19 and 36

38	limit 37 to (english language and yr="2007 -Current")

**Database: Cochrane Library all databases (via Wiley)**

**Date searched: 29^th^ November 2023**

#1	MeSH descriptor: [Cerebrovascular Disorders] this term only

#2	MeSH descriptor: [Basal Ganglia Cerebrovascular Disease] explode all trees

#3	MeSH descriptor: [Brain Ischemia] explode all trees

#4	MeSH descriptor: [Carotid Artery Diseases] explode all trees

#5	MeSH descriptor: [Intracranial Arterial Diseases] explode all trees

#6	MeSH descriptor: [Intracranial Embolism and Thrombosis] explode all trees

#7	MeSH descriptor: [Intracranial Hemorrhages] explode all trees

#8	MeSH descriptor: [Stroke] this term only

#9	MeSH descriptor: [Brain Infarction] explode all trees

#10	MeSH descriptor: [Vertebral Artery Dissection] 5 tree(s) exploded

#11	stroke or cerebrovasc* or (brain NEXT vasc*) or (cerebral NEXT vasc*) or cva* or apoplex*

#12	(brain* or cerebr* or cerebell* or vertebrobasilar or hemispher* or intracran* or intracerebral or infratentorial or supratentorial or MCA or "anterior circulation" or "posterior circulation" or "basal ganglia") NEAR/5 (ischemi* or ischaemi* or infarct* or thrombo* or emboli*)

#13	(brain* or cerebr* or cerebell* or intracerebral or intracran* or parenchymal or intraventricular or infratentorial or supratentorial or "basal ganglia") NEAR/5 (haemorrhage* or hemorrhage* or haematoma* or hematoma* or bleed*)

#14	{OR #1-#13}

#15	MeSH descriptor: [Palliative Care] this term only

#16	MeSH descriptor: [Palliative Medicine] explode all trees

#17	MeSH descriptor: [Hospices] explode all trees

#18	MeSH descriptor: [Terminal Care] this term only

#19	MeSH descriptor: [Death] explode all trees

#20	dying

#21	end of life

#22	end of days

#23	last days of life

#24	final days of life

#25	last month of life

#26	palliat*

#27	terminal NEAR/6 care

#28	bereave*

#29	(death or die or died) NEAR/6 (sudden* or unexpected* or unanticipated or "30 days" or "thirty days" or month)

#30	advance care plan or "advance care planning" or "advance care plans"

#31	advance* NEAR/1 statement*

#32	document* NEAR/3 (preference* or wish*)

#33	{OR #15-#32}

#34	#14 AND #33

#35	#14 AND #33 with Cochrane Library publication date from Jan 2007 to present

**Database: CINAHL Ultimate (EBSCOhost)**

**Date searched: 29^th^ November 2023**

S1	(MH "Cerebral Ischemia+")

S2	(MH "Basal Ganglia Cerebrovascular Disease+")

S3	(MH "Carotid Artery Diseases+")

S4	(MH "Cerebral Ischemia+")

S5	(MH "Cerebral Vasospasm")

S6	(MH "Intracranial Arterial Diseases+")

S7	(MH "Intracranial Embolism and Thrombosis")

S8	(MH "Intracranial Hemorrhage+")

S9	(MH "Stroke")

S10	(MH "Vertebral Artery Dissections")

S11	(MH "Stroke Patients")

S12	(MH "Stroke Units")

S13	stroke or cerebrovasc* or "brain vasc*" or "cerebral vasc*" or cva* or apoplex*

S14	(brain* or cerebr* or cerebell* or vertebrobasilar or hemispher* or intracran* or intracerebral or infratentorial or supratentorial or MCA or "anterior circulation" or "posterior circulation" or "basal ganglia") N5 (isch*emi* or infarct* or thrombo* or emboli*)

S15	(brain* or cerebr* or cerebell* or intracerebral or intracran* or parenchymal or intraventricular or infratentorial or supratentorial or "basal gangli*") N5 (haemorrhage* or hemorrhage* or haematoma* or hematoma* or bleed*)

S16	S1 OR S2 OR S3 OR S4 OR S5 OR S6 OR S7 OR S8 OR S9 OR S10 OR S11 OR S12 OR S13 OR S14 OR S15

S17	(MH "Palliative Care")

S18	(MH "Hospice and Palliative Nursing")

S19	(MH "Hospices")

S20	(MH "Terminal Care")

S21	(MH "Death+")

S22	dying

S23	"end of life"

S24	"end of days"

S25	"last days of life"

S26	"final days of life"

S27	"last month of life"

S28	palliat*

S29	terminal N6 care

S30	bereave*

S31	(death or die or died) N6 (sudden* or unexpected* or unanticipated or "30 days" or "thirty days" or month)

S32	"advance care plan*"

S33	"advance* statement*"

S34	document* N3 (preference* or wish*)

S35	S17 OR S18 OR S19 OR S20 OR S21 OR S22 OR S23 OR S24 OR S25 OR S26 OR S27 OR S28 OR S29 OR S30 OR S31 OR S32 OR S33 OR S34

S36	S16 AND S35 Published Date: 20070101-; English Language

**Database: PsycINFO (ESBCOhost)**

**Date searched: 29^th^ November 2023**

S1	DE "Cerebrovascular Disorders"

S2	DE "Cerebral Hemorrhage"

S3	DE "Cerebral Ischemia"

S4	DE "Cerebral Small Vessel Disease"

S5	DE "Cerebrovascular Accidents"

S6	DE "Subarachnoid Hemorrhage"

S7	DE "Brain Damage"

S8	stroke or cerebrovasc* or "brain vasc*" or "cerebral vasc*" or cva* or apoplex*

S9	(brain* or cerebr* or cerebell* or vertebrobasilar or hemispher* or intracran* or intracerebral or infratentorial or supratentorial or MCA or "anterior circulation" or "posterior circulation" or "basal ganglia") N5 (isch*emi* or infarct* or thrombo* or emboli*)

S10	(brain* or cerebr* or cerebell* or intracerebral or intracran* or parenchymal or intraventricular or infratentorial or supratentorial or "basal gangli*") N5 (haemorrhage* or hemorrhage* or haematoma* or hematoma* or bleed*)

S11	S1 OR S2 OR S3 OR S4 OR S5 OR S6 OR S7 OR S8 OR S9 OR S10

S12	DE "Palliative Care"

S13	DE "Hospice"

S14	DE "Terminally Ill Patients"

S15	DE "Death and Dying"

S16	DE "Sudden Death"

S17	dying

S18	"end of life"

S19	"end of days"

S20	"last days of life"

S21	"final days of life"

S22	"last month of life"

S23	palliat*

S24	terminal N6 care

S25	bereave*

S26	(death or die or died) N6 (sudden* or unexpected* or unanticipated or "30 days" or "thirty days" or month)

S27	"advance care plan*"

S28	"advance* statement*"

S29	document* N3 (preference* or wish*)

S30	S12 OR S13 OR S14 OR S15 OR S16 OR S17 OR S18 OR S19 OR S20 OR S21 OR S22 OR S23 OR S24 OR S25 OR S26 OR S27 OR S28 OR S29

S31	S11 AND S30 Publication Year: 2007-; English
