# Supplementary material for: Experiences, barriers and enablers of stroke end-of-life care: a systematic review with mixed-methods synthesis
Source: BMC Palliat Care. 2026 Jul 27;25:317. doi: 10.1186/s12904-026-02211-z (PMC13689126; doi:10.1186/s12904-026-02211-z)
Supplement: Supplementary file 2 [file 12904_2026_2211_MOESM2_ESM.docx]

| **Author**  **Country** | **Aims** | **Design** | **Clinical Setting** | **Participants** | **Main Results** |
| --- | --- | --- | --- | --- | --- |
| **Qualitative studies** | | | | | |
| de Boer at al. ^21^  Nether-lands | To elicit the perspective of relatives of severe stroke patients with regard to the decision-making process in the acute phase in order to understand how they participate in treatment decisions. | Exploratory qualitative approach guided by the principles of grounded theory. | 3 academic and 1 non-academic hospital. | N=15 Relatives of patients who had suffered from a severe stroke. | Four categories reflecting relatives’ experiences:   1. Making ‘choices’ under time pressure 2. The feeling of ‘who am I’ to decide 3. Reluctance in saying ‘let him die’   Coping with unexpected changes |
| Eriksson et al. ^22^  Sweden | To study the ethical dilemmas, approaches, and consequences these issues create among healthcare professionals regarding dying patients with stroke in acute stroke units. | Focus groups, individual interviews and questionnaire. Analysed using content analysis. | One university hospital and two general hospitals with stroke units | N=36 Multi-disciplinary healthcare professionals | 1. Two categories of ethical dilemmas: a) Non-decisions b) Problems holding to the decision 2. Communication barriers within the MDT around prioritising End-of-life care. |
| Gardiner et al. ^8^  UK | To explore the perspectives of specialist stroke health professionals regarding the provision of palliative and end-of-life care in UK stroke units. | Qualitative focus groups and individual interviews. Analysed using thematic analysis. | UK specialist stoke units | N=66 Health professionals | Three themes:   1. Palliative care as a recognised component of stroke care 2. Uncertainty in making transitions to palliative care 3. Issues integrating acute stroke care and palliative care. |
| Lloyd et al. ^23^  India | To describe treating doctors’ perspectives on the palliative needs of stroke patients in India. | Qualitative approach using semi-structured interviews and thematic analysis. | A tertiary care hospital. | N=17 Qualified doctors in medicine, neurology and rehabilitation departments. | 8 themes:   1. Functional disability 2. Physical burden 3. Psychological needs 4. Social issues 5. Caregiver burden 6. Counselling the caregiver-an unmet need 7. Spiritual needs 8. Issues at the end of life care |
| Payne et al. ^10^  England | To explore patients’ and family members’ accounts of their experiences and views of end-of-life care and to identify barriers and opportunities to improve care. | Cross-sectional exploratory design using semi-structured Interviews. Constant comparative method to develop coding framework for analysis. | Two district general hospitals with specialist palliative care teams. | N=28 Stroke patients and N=25 family members. | Main themes:   1. Communication between family and health professionals 2. Information provision – being involved in medical decisions 3. Facing uncertainty of recovery 4. End of life preferences. |
| Rejnö, et al. ^24^  Sweden | To describe experiences of members of stroke teams working in stroke units of ethical problems and how the teams manage the situation when caring for patients faced with sudden and unexpected death from stroke. | Focus group interviews analysed using interpretive content analysis. | Stroke units in four hospitals providing both acute and rehabilitative stroke care. | N=19 Team members caring for patients who are dying due to acute stroke. | Three partially overlapping themes:   1. To give information when conditions are unstable 2. To decide on care when the situation is changing 3. To support when uncertainty prevails   Uncertainty created by suddenness and unexpectedness ran like a ‘red thread’ through all the themes and linked them together. |
| Rejnö, et al. ^25^  Sweden | To illuminate the experiences of next-of-kin from the sudden and unexpected death of a relative from acute stroke. | Narrative interviews, analysed using a thematic narrative approach. | Interviews conducted in the participants’ homes and the author’s office. | N=12 Next-of-kin. | The findings illuminate various facets of the next-of-kin’s experiences of having a relative who died a sudden and unexpected death from stroke.  Three themes:   1. Divided feelings on sudden and unexpected death 2. Perception of time and attention when keeping vigil 3. Contradictions and arbitrary memories when searching for understanding. |
| Rejnö, et al. ^27^  Sweden | To describe how nurses experience the patient’s death and dying when patients are afflicted with acute stroke and whose lives cannot be saved. | Descriptive design with a hermeneutical approach. | Acute and rehabilitative stroke units in three county hospitals. | N=10 Registered Nurses. | The main theme of Sudden death – the unexpected force has been described by the three subthemes:   1. Death comes unexpectedly and without warning to the patient 2. The relatives are at the mercy of the unexpected 3. The nurses find themselves in demanding situations. |
| Rejnö and Berg ^26^  Sweden | To illuminate obstacles and possibilities perceived by stroke team members in using strategies for handling ethical problems when caring for patients afflicted by sudden and unexpected death caused by stroke. | Individual interviews. Deductive and inductive content analysis. | Stroke units of two associated county hospitals. | N=15 Stroke team members. | The findings show the obstacles and possibilities the stroke team members perceive exist in using the nine strategies for handling ethical problems.  Not-preferred strategies:   1. Continue treatment (e.g. drip) 2. Decide against the will of next-of-kin 3. Withhold decisions   Preferred strategies:   4. Change decisions made if the situation changes 5. Discuss with next-of-kin or the team 6. Inform next-of-kin 7. Meet requests of next-of-kin 8. Support next-of-kin 9. Unanimity with next-of-kin or within the team. |
| Rejnö, et al. ^28^  Sweden | To deepen the understanding of stroke team members’ reasoning about truth-telling in end-of-life care due to acute stroke with reduced consciousness. | Individual interviews with combined deductive and inductive content analysis. | Stroke units of two associated county hospitals. | N=15 Acute and rehabilitation stroke unit teams. | Not to do harm could be described as a common thread, connecting all the categories in a united theme.  The findings are presented in two categories and five subcategories.   1. Truth above all  - A value in itself - To establish trust  2. Hide truth to protect  - Not add extra burden in the sorrow - Awaiting the timely moment - Not being a messenger of bad news . |
| Rejnö and Berg ^29^  Sweden | To illuminate the communication and its meaning in unexpected sudden death with stroke as an example, as experienced by stroke team members and next-of-kin. | Secondary analysis of data from four previous interview studies. | County hospitals which provided both acute and rehabilitative stroke care. | Study A–C: N=27 stroke team  Study D: N=12 next- of-kin of patients who had died due to acute stroke during hospital stay. | Overarching theme - foundation for dignified encounters in care built-up by six themes illuminates the meaning of communication in unexpected sudden death by stroke.   1. Creating a relationship is crucial for communication between next of kin and the team members 2. Information enabling understanding 3. Ground for unanimity 4. Personalise the patient 5. Preconditioned for decisions about care 6. Giving and experiencing support. |
| Rogers & Addington-Hall ^31^ | To understand how care is provided and  experienced at the end of life in stroke, and to begin to identify what role  (if any) palliative care might have in providing this care. | 6 months data collection. Non-participant observation. Interviews with patients’ relatives and healthcare professionals prior to and following death.  Constant comparative approach to analysis. | One acute stroke unit, inner city teaching hospital. | N=22 patients (age range 58-106 years). Eleven women.  Staff and relative participants not reported. | Themes:   1. Impact of time to death 2. General nursing care  - Artificial feeding and hydration   Complexity in decision-making. Relatives and staff are motivated by different factors in making decisions on feeding.   3. Communication  - Prognostication. Decisions on whether a patient is likely to die early after stroke the most challenging for staff - Care in last hours   Patients suffering a severe stroke may be less well cared for  in non-specialist environments, and this needs further investigation. |
| Su et al. ^30^  China | To explore perspectives of primary caregivers in providing palliative care for Chinese patients with stroke. | Individual, semi-structured interviews. Thematic analysis. | Geriatric unit of a major hospital. | N=17 (88% female) Healthcare professionals who had cared for a patient with stroke for at least 1month and provided  full-time bedside care. | Promoting comfort was the overarching theme  5 sub-themes were:   1. Meeting physical care needs 2. Ensuring communication 3. Psycho-emotional care   2 additional domains critical in the Chinese context:   4. Providing cognitive stimulation; 5. Avoiding discussions on death and dying.   The concept of death appears negative; discussion about death was considered disrespectful. Physical symptom burden and psychological distress are prominent among patients dying from stroke.  Despite the uncertainty in stroke prognosis, the life threatening attributes, high care needs, and disease burden are comparable with other illnesses with a clear dying trajectory.  The core attributes of PC (physiological care needs, ensuring communication, providing psycho-emotional care; stimulating cognitive reaction) should be incorporated into stroke PC. Advanced care planning and specialist palliative care should be used at an early stage. |
| **Quantitative studies** | | | | | |
| Blacquiere et al. ^32^  Canada | To understand how the guidelines are translated into clinical management, and to show that an organised approach to management of patients with devastating stroke leads to better end of life care. | Retrospective chart examination. Information was collected: FSSS, physicians orders, investigations, vital signs, nursing care, feeding and fluids, family discussions about care.  Descriptive statistics only. | Acute Stroke Unit (ASU) of the QEII Health Sciences Centre (QEII HSC) in Halifax, Nova Scotia | N=104 Stroke patients admitted 2003-04.  60.6% Female  Median age 80 years  Stroke severity FSSS (Functional Stroke Severity Scale) : 0-6 – 12.5%  7-10 – 86.5%  Unknown – 1.0% | 90.4% were palliated.  Median time from admission to palliation was 3.6 days  Median time from admission to death was 8.5 days.  Most had vital signs (98.9%), investigations (100%) and non-palliative medications (95.7%) stopped, and had nasogastric feeding (96.8%) and intravenous fluids (87.2%) withdrawn or never begun. 93.6% were treated with morphine and 81.9% with scopolamine.  Concerns raised by family members centred around hydration and feeding (45.7%), doubts about palliative care (27.8%) and patient comfort (18.2%).  Families had interactions with staff about: provision of information (57.7%), stroke prognosis (49.0%), changing to palliative care (69.2%), provision of comfort (42.1%), provision of medication (34.6%), cessation of feeding (44.2%). Within these interactions (N=104), some were designated conflicts: provision of information (13.3%), stroke prognosis (2.0%), changing to palliative care (27.8%), provision of comfort (18.2%), provision of medication (13.9%), cessation of feeding (45.7%). |
| Blacquiere et al. ^33^  Canada | To determine how families of acute stroke patients perceived palliative care after stroke. | Telephone Interview using validated questionnaire for palliative research.  Correlational analysis of psychometric properties, factor analysis and internal reliability testing. | University of Ottawa Neurology and Neurosurgery services. | Patient’s next- of-kin (N=15, 55% response rate):  (child of patients (86.67%), spouses (6.67%) and friends (6.67%)).  Patient details (N=15):  Female 46.7%  Median age 80 years  Stroke type:  Ischemic N=10 (66.7%)  ICH/SAH N=5 (33.3%)  Median NIHSS =20 | 53.3% independent before hospitalisation.  Power of attorney (healthcare) assigned N=12 (80.0%). Advanced care planning before stroke N=7 (46.7%).  Median time in days:  Admission to discussion of palliation 1.2  Admission to palliation 2.8  Stroke onset to death 9.9  Palliation to death 5.0  Feeding/fluids  IV fluids stopped N=14 (93.3%)  NG feeding stopped/never started N=15 (100%)  Domain/problem scores, 0-1 with higher scores represent less satisfaction:   - Feeding, hydration and communication 0.41 - Coordination of care 0.26 - Attention to family needs 0.27 - Focus on individual 0.24 - Advance care planning 0.23 - Adequate anxiety/depression treatment 0.33 - Adequate dyspnoea treatment 0.22 - Adequate analgesia given 0.17 - Adequate information to family regarding analgesia 0.31 - Adequate family understanding of analgesia 0.25   Overall satisfaction with aspects of palliative care 0-10, higher score represents greater satisfaction:   - Overall 9.14 - Emotional support for families 8.79 - Ensured dignified death 9.14 - Adequate symptom control 8.79 - Respectful medical care 9.14 - Communication with patient 9.21 |
| Burton et al. ^34^  England | To identify the palliative care needs in a consecutive cohort of acute stroke patients. | Prospective study  Sheffield Profile for Assessment and Referral to Care (SPARC) questionnaire/ interview.  Descriptive statistics. Predictors of need identified via multiple regression and explored using General Linear Modelling techniques. | One UK city, all acute stroke patients admitted to two hospitals. | N=191 acute stroke patients  47.6% female  98.4% had 1+ comorbidities  25.7% aged <65 years | Nearly 80% experienced some form of communication problem.  Fatigue-related problems were prevalent, with over 50% reporting moderate to significant problems with ‘feeling weak’, ‘feeling tired’ or ‘being sleepy during the day’. 70% reported problems with ‘feeling everything’s an effort’  Approximately 50% reported problems with ‘pain’, ‘memory loss’, ‘headache’, ‘restlessness’ or ‘bladder problems’.  40% had moderate to significant ‘fear of falling’. Approximately 50% experienced some form of psychological distress such as ‘anxiety’, ‘low mood’, ‘confusion’, ‘poor concentration’ and ‘loneliness’. Prevalence of religious or spiritual concerns was low. Approximately 25% had concerns about death or dying.   - Approximately 66.6% had concerns relating to dependence and disability. - Over 50% were worried about the effects of their stroke on others within their support network. - 25% felt that they needed more help than their family could provide.   Patient dependence was a significant predictor of scores on physical, psychological, and religious and spiritual SPARC domains, and with female gender and age, on the dependence and disability domain.  Higher levels of dependence and female gender were associated with greater concerns about dependence and disability. Patient dependence, female gender and the number of co-morbidities were significant predictors of scores on the SPARC family and social domain.  A model confirmed the significant main effects of the Barthel index score and age in predicting SPARC score. The model indicated a significant three-factor interaction between Barthel Index, Age and Co-morbidities. |
| Kamdar et al. ^35^  USA | To determine if early integration of palliative care consultations was feasible and would impact understanding, decision-making and emotional support to patients and surrogate decision-makers.  To evaluate the long-term impact of palliative care assessment on development of post-traumatic stress disorder (PTSD) amongst patients or surrogate decision-makers. | Single centre, prospective pilot randomised control study.  Questionnaire on Communication (QoC), Decisional Conflict Scale (DCS), and Hospital Anxiety and Depression Scale (HADS). DSM-5 (PCL- 5)  Descriptive and inferential statistics for demographics.  Linear mixed effects modelling for association between intervention and patients’ or surrogate’s scores. | Neurocritical care unit (NCCU). | Patients (N=22) with moderate to severe ischemic and haemorrhagic stroke and their surrogate decision makers.  Median age 65.5 years  Female 45.5%  Haemorrhage 27.3%  Ischemic 59.1%  Ischemic 13.6%. | Control arm: standard intensive care  Intervention arm: additional early palliative care consultation within 72 hours of hospitalisation.  There were no significant differences in the QoC and DCS scores between the groups and across timepoints.  Significant improvement in scores was seen in the total HADS score (p=0.043) and PCL5 score (p=0.033) at 3 months following intervention. |
| Kerever et al. ^36^  France | To assess the potential influence of nurses on physician’s decisions made to withhold and/or withdraw treatments for stroke patients at the end of life. | Secondary analysis nested within the TELOS French national survey: based on a physicians' self-report questionnaire and on a I-Score which was linked to nurses' involvement.  Physician's responses were evaluated to assess the potential influence of nurse's involvement on physician's choices during an end-of-life decision  Descriptive and inferential statistics. | 49 stroke units among the 100 listed in the TELOS survey. | Head physician in charge of each stroke unit.  Ward physicians/ senior neurologists.  N=126 physicians consented to take part,  120 physician questionnaires could be analysed.  44% female.  Approx 50% and 37% of physicians reported 10+ years of experience in neurology and stroke management respectively. | 64% took part in specific “team meetings” dedicated to end of life decisions and patients' outcomes.  The median I-Score was 10.  Neurologists that more take into account nurses' opinions  withheld active treatment more often (98% vs. 88%, p = 0.04), withdraw nutrition and artificial hydration more often (59% vs. 39%, p = 0.04), and to prescribe analgesics and hypnotics to potentially lethal doses more frequently (70% vs. 48%, p = 0.03) compared to neurologists that did not confer with nurses on end of life decisions.  Physicians that were more involved with nurses when making end of life decisions reported that decisions were often made during specific round-table discussions dedicated to severe-stroke patients (24% vs. 11%, p = 0.02). This occurred more frequently during medical or unit roundtable discussions (58% vs. 35%, p = 0.004) and less often at a patient's bedside (25% vs. 49%, p = 0.02).  Physicians who were more involved with nurses during end of life decisions responded that, when do not resuscitate orders were decided upon, the “family's opinion was important” (82% vs. 64%, p = 0.047) and that it was less often “necessary to decide” (12% vs. 30%, p = 0.047).  A potential disagreement with the family happened more often in the physician group that seem to involve nurses (12% vs. 5%, p = 0.046). These physicians said it was important that a physician from another unit added an external viewpoint during these meetings on end of life decisions (80% vs. 59%, p = 0.02).  For do not resuscitate orders, 22% thought the family's opinion was necessary for decision-making, 73% thought it was important and 5% thought it was not necessary.  69% considered it important that physician from another unit, or the palliative-care team are present during an end of life decision. |
| Khosravani et al. ^37^  Canada | To investigate the experiences and perceived barriers of palliative care (PC) for patients with acute severe stroke | Survey (descriptive, cross-sectional, web-based self-administered)  Three sections related to stroke physician characteristics, practice attributes, and opinions about palliative care.  Descriptive statistics, univariate, and multivariate regression analysis were performed | Acute stroke care | Stroke physicians in Canada who engage in acute severe stroke care.  N=69  41% Female  Percentage of physician practice involved in care of patients with acute severe stroke 25% or less: 25 %, 25-50%: 25%, 50-75%: 23% and 75% or more: 28%. | In order of importance for the multivariate model (on a standardized scale):   - Stroke physicians reported greater PC team involvement when there was more agreement that the family had adequate education regarding PC care for stroke patients - When the stroke physician was more often involved with discharge planning with PC services, when the stroke physician had a higher proportion of practice in stroke care - When the physician has a stroke fellowship - When there is more agreement that a PC specialist is the best person to coordinate the PC needs of patients with acute severe stroke - When the stroke physician practices in a teaching hospital   Barriers to integrating PC early in the trajectory of acute severe stroke.  1=Disagree strongly, 2=Disagree, 3=Neutral, 4=Agree, and 5=Agree strongly. Median (IQR)  1.Uncertainty in the patient's prognosis: 4, (4.4)  2.Not being able to access a palliative care specialist when needed: 3, (2.5)  3.Confusion surrounding role of the palliative care specialists 3, (2.4)  4.Not being aware of what a palliative care specialist can contribute 3, (2.4)  5.Substitute Decision Makers (SDMs) do not accept the palliative philosophy of care early in the trajectory of acute severe stroke: 4, (4.5)  Consultation to PC team:   - Stroke physicians reported that the PC team was involved “sometimes” for patients with acute severe stroke (mean=2.9, SD=0.8). - When the stroke physicians consulted the PC team, they were doing so most often for discharge planning (mean=4.1, SD=1.1) followed by administration of EOL care (mean=3.9, SD=1.2). - The PC team was consulted “sometimes” for grief and bereavement support (mean=3.6, SD=1.2 and 1.4 respectively), treating pain and other physical symptoms (mean=3.3, SD=1.2) and managing psychological symptoms (mean=3.1, SD=1.3), and “rarely” for prognostication (mean=2.3, SD=1.2).   Stroke physician providing PC care to patients with acute severe stroke:   - Stroke physicians were “often” to “always” involved with prognostication (mean=4.6, SD=0.6) and discussing goals of care with patients or their substitute decision maker (SDM) (mean=4.4, SD=0.8), and “sometimes” involved with treating pain (mean=3.8, SD=1.1), psychological symptoms (mean=3.4 SD=1.0) and discharge planning (mean=3.0, SD=1.3). - Stroke physicians were “rarely” to “sometimes” involved with administering EOL care (mean=2.7, SD=1.4), grief support (mean=2.7, SD=1.3) and bereavement (mean=2.5, SD=1.2).   Stroke physician management of acute severe stroke patients   - Stroke physicians endorsed that the PC specialist was best positioned to coordinate the PC needs of their patients (mean=4.0, SD=1.0) and agreed that dying patients were best managed on a PC unit (mean=3.9, SD=1.1). - Agreement on benefit from PC education (mean=3.9, SD=1.1) and that they should coordinate the care of their own patients at all stages (mean=3.7, SD=1.1). - Stroke physicians felt neutral about PC specialists not having enough understanding to counsel patients and their families regarding prognosis (mean=3.0, SD=1.4). - Stroke physicians provided insight into some of the challenges associated with the provision of PC such as sufficient time to spend with the family (mean=2.6, SD=1.2) and having adequate training with PC in their stroke fellowship (mean=2.2, SD=1.2). |
| Markovitz et al. ^38^  USA | To assess surrogate perceptions of quality of End-of-life care in stroke and explore factors associated with quality | Cross-sectional analysis of interviewer-administered survey (quality of end-of-life care, QEOLC scale)  The univariate association between prespecified patient and surrogate factors and dichotomized QEOLC score was explored via Logistic regression fit using generalized estimating equations.  A sensitivity analysis investigated the association between ordinal QEOLC and potential predictive factors using the Cochran–Armitage trend test. | Population-based study | Surrogate (N=79) decision-makers for patients who died from stroke (n=66)  Surrogate details:  Median age 59 (16) years  Female 72.2%  Spouse 22.2%,  Child of patients 65.8%,  Other 11.4% | Informal discussions about advance care planning: Yes 88.6%, No 11.4%.  Formal advance directive: Yes 38%, No 62%.  The overall QEOLC (scored 0-10) was generally high (median 8.3, quartiles 6.1, 9.6).  82.3% of surrogates had high self-reported confidence to serve as a surrogate, 16.5% had low.  No hypothesized factors were associated with QEOLC score, including demographics, stroke type, location/timing of death, advance directives, health literacy, or understanding of patient wishes.  The sensitivity analysis investigating ordinal QEOLC found no association with patient or surrogate factors other than NIHSS, with more severe strokes associated with lower QEOLC (correlation coefficient = 0.25, p = 0.03). |
| Reinink et al. ^39^  Netherlands | To assess the experience of family members about symptoms during the terminal phase and the ‘quality of dying’ in patients who deceased in a stroke unit; and opinion of nurses of quality of dying of same patients. | Structured interviews/ validated measures  QODD-ICU completed by the patient's nurse and relatives  Inferential and descriptive stats. Linear regression to analyse relation of patient and clinical characteristics on relative’s experience of dying. | Single-centre stroke unit | Relatives (N=59) and nurses (N=56) who provided End-of-life care. | Relatives were generally satisfied with:   - Quality of dying (median score 8; IQR 3). - Care provided by nurses (9; 8–10) and doctors (8; 7–9). - Breathing difficulties reported by 46% of the relatives. - Unsatisfactory experiences were related to feeding (69% unsatisfactory), inability to say goodbye to loved ones (51%), appearing not to have control (47%), and not retaining a sense of dignity (41%), r ‘appeared to breath comfortably’ (38% unsatisfactory). - Communication, information and support by the medical personnel (8; 8–9). - Median length of the end-of-life was 14 h (IQR 4–31). This was too short for 9 % of relatives, 53% neither too long or short, 27% too long.   “In general, reports of nurses were very much in line with the reports of relatives” |
| Reisinger et al. ^40^  Austria | To assess whether having early and close cooperation with specialist palliative care (PC) services has an impact on stroke patients’ families satisfaction.  To evaluate decision-making in the context of limiting life-sustaining therapies (LST) in severe stroke | Prospective study using questionnaire (FS-ICU 24) on family satisfaction with decision-making.  Descriptive statistics and prevalence reporting only | Acute inpatient stroke unit | Family (N=17) of patients with severe ischemic stroke; and stroke team.  59% female.  Child of patient 71%  18% siblings  12% other.  N=18 stroke team members completed the questionnaire59% nurses, 41% physicians. | Specialist PC consultation took place 3 days (±3 days) after stroke and, in 95% of cases, was conducted face-to-face.  On average, two contacts (±2; mean ± SD) occurred during the hospital stay. The PC physician was involved in all and the PC nurse in 35% of cases.  The focus of PC contact was primarily on the assessment and therapeutic advice in symptom management (70%), assistance in transfer to specialist PC services (65%), communication and support for families (55%), and decision-making (30%).    Family satisfaction was scored excellent-good by:   - 97% for care for patient - 100% for skills team - 97% for care for family - 96% for information - 100% for amount of care   Family decision making:   - 89% were somewhat or very included in the process, 6% felt very excluded, 6% were neutral - 88% felt very/supported, 12% were neutral - 82% felt very in control/some control, 18% neutral   Stroke team:  Physicians reported that nurses were involved in 71% of cases, whereas nurses felt involved in 40%.  Satisfaction with decisions (physicians 100%, nurses 90%) and communication (physicians 100%, nurses 80%) was high. Of physicians, 14% felt insufficiently trained and insecure in situations dealing with limiting LST, whereas of nurses, 30% felt insufficiently trained and 10%, insecure.  The topic of limiting LST was considered important by both physicians and nurses (100% each) and their need for information proved high (physicians 100%, nurses 90%).  Physicians' challenges for limiting LST were: communication with patients/family, 57%, ethical concerns 57%, legal concerns 43% and religious concerns 10%.  Nurse’s challenges for limiting LST were: communication with patients/family 60%, ethical concerns 20%, legal concerns 20%, communication with other professions 20%, religious concerns 10%, communication within profession 10%. |
| Rogge et al. ^41^  Germany | To investigate the influence of neurologists’ previous professional experience on decision pathways in end-of-life decision-making in stroke cases. | A fictional case vignette.  Questions on prognosis, interpretation of an advanced directive, withdrawing life-sustaining treatments and information on prior rehabilitation experience.  Likert scales were used for Q1 and 2.  Q1: Unlikely (1)→Likely (10)  Q2: Very uncertain (1)→Very certain (10)  Q3 Correct or Incorrect  Q4 Yes or no  Descriptive, inferential statistics and correlation to compare decisions across patient demographics. | All hospitals with certified stroke units listed by the German Stroke Society. | Neurologic specialists (N=74; response rate: 14.8%) working in stroke units or in the ICU.  Mean age 45.9 years (SD 5.8)  Female 24%  83% had ≥10 years' acute medicine experience. | Sample analysed N= 70. 4 returned surveys were omitted from analysis.  77% opted for the withdrawal of life-sustaining therapies. This decision was not affected by age, gender, or length of clinical experience. Neurologists with experience in rehabilitation medicine tended to estimate a more positive prognosis than neurologists without, but this result was not significant (p=.13).  Neurologists with experience in rehabilitation medicine opted significantly more often (31.8%) for continuing life-sustaining treatments than neurologists without such experience (8.7%, p=.04).  When asked how likely respondents considered the probability for the patient to regain consciousness and ability to communicate (Q1, mean 3.6 SD 2.2, range 1-8). Most participants ranked their confidence of this estimate on the certain side of the spectrum (Q2, mean 7.4, SD 1.79, range 2–10. The correlation between these questions was significantly negative, r(70)=−.30, p=.01. There were no significant correlations between Q1 or Q2 with gender, age or years of experience.  N=56, 80% of neurologists assumed that insertion of a feeding tube and tracheostomy would not correspond with the will of the fictive patient (Q3). N=54, 77% of clinicians would not argue for the execution of the procedures in this case (Q4).  There was no significant difference between neurologists with and without rehabilitation regarding prognosis (U(23,44)=393.5, p=.13). Neurologists without rehabilitation experience opted significantly more often against life-sustaining therapies (χ2 (1, N=67)=4.44, p=.04). |
| Skolarus et al. ^42^  USA | Explore advanced care planning engagement and efficacy among patients with stroke/transient ischemic attack (TIA). | Cross-sectional structured telephone interviews  Survey assessed 4 critical advanced care planning elements. Responses were dichotomised as “already done” vs not.  Patients and surrogates were queried about life-prolonging treatment preferences.  Descriptive stats and Cohen’s kappa to test agreement between patient and surrogate. | Acute stroke centre. | Patients (N=50) with stroke/TIA admitted to stroke unit  Mean age 68.1 years (SD=12.8) 54% were women  17% reported limited health literacy.  Median modified Rankin Score = 0 (IQR 1). | N=23 (46%) participants with stroke/TIA reported designating a surrogate decision-maker.  N=25 (50%) had discussed their wishes with a surrogate.  N=5 (10%) had discussed their wishes with a provider.  N=17 (34%) had completed an advanced directive.  N=17 (34%) surrogate decision-makers participated in the study.  Overall mean 5-point Advance Care Planning Engagement Survey score was 3.3 (SD=1.1).  Eight (35%) of those with a durable power of attorney and N=4 (24%) of those with an advance directive reported patient portal deposition.  14% of patients with stroke/TIA discussed their wishes with a surrogate decision-maker after their stroke/TIA.  Limiting to those patients with stroke/TIA who identified a surrogate, for the severe pain LPT scenario, N=4 (25%) reported wanting to stop/reject treatments, and 63% of patient/surrogate pairs agreed (N=16, Kappa=0.32, P=0.08). For the severe disability LPT scenario, N=9 (56%) reported wanting to stop/reject treatments and 44% of patient/surrogate pairs agreed (N=16, Kappa=−0.13, P=0.43). |
| Zwicker et al. ^43^  Canada | To improve the comfort/self-perceived efficacy of nurses caring for patients at the end of life after stroke. | Model for Improvement framework.   1. Pre-education survey and Pre-Palliative Care in Stroke Comfort Scale 2. 10 Education sessions 3. Post- Palliative Care in Stroke Comfort Scale.   Pre and post education scores were compared using a paired, two-tailed t-test. | A tertiary care hospital. | Pre-education survey completed by N=54 nurses.  Education delivered to N=71 (N=54 nurses and N= 12 allied health professionals, N=5 did not identify their professional designation)  N=17 nurses completed post-training survey. | Primary outcome: improvement in the Stroke End-of-Life Care Comfort Scale for nurses.  There was a significant improvement in the Stroke End-of-Life Care Comfort Scale score (p=0.00004) 3–4 weeks after the education session compared to the score before the session.  There was no significant difference between the score before the education session of the 17 nurses that completed the score after 3 weeks compared to the nurses that did not. |
| **Mixed methods** | | | | | |
| Burton and Payne ^18^  UK | To develop a programme theory to explain the integration of palliative and acute stroke care around the needs, experiences and preferences of patients and family members. | This paper synthesises three sources of data. The other studies (Burton, Payne et al. 2010) (Payne, Burton et al. 2009) have been extracted individually. This data reports on the data synthesis of the third study (group interviews). Analysis using programme theory. | Three hospital-based stroke services in the north of England. | N=29 stroke unit staff. | Our data suggest that a programme theory that integrates palliative and acute stroke care should attend to six key mechanisms.  Cognitive mechanisms:   1. Legitimacy of palliative care 2. Individual capacity   Behavioural mechanisms:   3. Engaging with family 4. The timing of intervention 5. Working with complexity 6. The recognition of dying. |
| Cowey et al. ^19^  Scotland | 1. To identify family and healthcare worker perceptions of the use of an end-of-life care pathway for patients who die after acute stroke.   To determine whether patients with fatal stroke judged to require an end-of-life care pathway differ from patients with fatal stroke who die without introduction of such a pathway (predictors of care pathway use). | Mixed methods approach, integrating qualitative interviews with quantitative case note review. Thematic analysis of interviews and data integration with quantitative data. | 4 acute stroke units (3 city teaching hospitals and 1 local general hospital). | N=17 Bereaved relatives and N=23 healthcare professionals. | Overarching themes related to End-of-life care:   1. Difficulty and uncertainty 2. Care quality   Other themes:   - Deciding is a series of steps - Prolonged dying - Active families - Proactive information-giving - Wondering about home or hospice care. |
| Rejnö, Danielson and Berg ^20^  Sweden | To illuminate stroke team members’ preferences and usage of ways to handle ethical problems and perceived hindrances in the care | Study-specific form and interviews. This study is a follow-up of a focus group study performed in 2009. | The study was carried out with team members from SUs in county hospitals. | N=15 Team members working in SU care and in the care of patients dying due to acute stroke. | Carers specified several hindrances that prevented them from using the strategies in the way they preferred and thereby made it impossible to work as they would wish. Eight hindrances were revealed as impeding the use of the strategies the way they were preferred.   1. Own shortcomings and personal characteristics 2. Knowledge and experience 3. Communication 4. Next of kin and their reactions influence and direct 5. Approaches to work/organisation/routines 6. Resources and priorities 7. Limitations and prerequisites inherent in the professional role 8. Conditions of reality |
